# Impotence

**Published:** 1881-01-20

**Authors:** Samuel W. Gross


### IMPOTENCE.

BY SAMUEL W. GROSS, M.D.

GENTLEMEN: We will consider this morning a subject that is very badly understood and treated. Impotence, or inability to perform the sexual act, may be relative or absolute; its treatment has been entirely empirical, and the sufferer discouraged by the pooh-poohs of one physician, goes to another, and so on indefinitely, until he falls into the hands of one who appreciates his condition, treats him properly, and cures him. In the Medical and Surgical Reporter of May 5, 1877, there is reported a paper I read before the County Medical Society, bearing the title "On Sexual Debility and Impotence, etc." In this article I divided such affections into four groups, based upon personal observation of eighteen cases, fifteen resulting from masturbation, and three from gonorrhœa. Since that time I have had seventy-nine more; the whole statistics show thirty to be the result of clap, sixty-six of masturbation, and one of excessive coitus. I asserted in my paper, that in sexual exhaustion certain lesions were always discoverable in the urethra; and with the additional experience of the last three years I fully corroborate the statements I published May 5, 1877.

What are the lesions present in impotence? We expect to find hyperæsthesia in all cases in the curve of the urethra, that portion which embraces the prostatic, membranous, and one inch anterior to the triangular ligament. Not infrequently a stricture is present. The groups of cases are arranged as follows: The first are those in which the erections are imperfect and the ejaculations are premature, but the sexual desire remains. Of eighty-seven cases which came under my care, sixty were the result of masturbation, and twenty-seven the consequence of gonorrhœa. This constitutes the most frequent group or class, ninety per cent. of all my cases having been of this kind. We have had several illustrations of premature ejaculation with imperfect erection, but with unabated desire, before you during the course.

At our last meeting, you will remember, I showed you a man who, for the last eighteen months, had been impotent, and though denying masturbation, presented such physical evidence as justified us in concluding that he was an onanist. This group is not only the most common, but also the most easily managed. In the article referred to, I say that the "condition known as spasmodic spermatorrhœa, or spermaspasmos, in which emissions occur simultaneously with erection, or after its partial subsidence," may be classed with the first group, because it is also due to increased excitability of the spinal cord. Here is a case: A clerk, aged thirty, brought me, on the 12th of March, a specimen of his urine to examine. He never had gonorrhœa, but masturbated from his 16th to his 21st year. For the past three years he has been impressed with the idea that his nervous habit had weakened him, and it was constantly on his mind. His genital organs were well developed; there was a constant sticky feeling at the meatus, and whenever he passed an evening with the lady upon whom he had fixed his affections, he had an erection with simultaneous ejaculation. There was hyperæsthesia of the prostatic urethra with a stricture, calibre 17,

6½ inches from the meatus. Thus two classes of cases seem to be due to increased reflex sensibility of the spinal cord.

The second group is where the desire is not abolished, but there is no erection, and coitus is impossible. Four such cases have come under my observation, two from clap and two from masturbation. In this variety of the affection the patient fails to get an erection, and intromission is therefore impossible. This condition is beautifully illustrated by case three in the article already ieferred to.

The third class of cases have neither ability nor desire, but there is superadded mental impotence, hypochondriasis, which is often beyond remedy. I have met with but two cases, and they resulted from masturbation. One case is briefly as follows: A student of medicine, aged 24, had masturbated from his 16th to his 22d year, and from that time had nocturnal seminal losses on an average twice a week. I saw him in May, 1875, when he told me that he had lost all desire, and had been unable to command an erection for three months. His mind was constantly dwelling on his trouble; he was the victim of sexual hypochondriasis; had a slight urethral discharge, which I demonstrated to him not to be spermatic fluid. I discovered a stricture, calibre 17 at 5¾, and found marked hyperæsthesia. The patient went with me to the sea-shore, and after three weeks of treatment his mental anxiety was calmed, and he had good erections. Upon his own responsibility he desired to test his sexual abilities, but with the erection had an almost instantaneous emission. This imprudent act voided all the good that had been accomplished, and he became a confirmed hypochondriac.

The first class of cases are those of diminished spinal reflex action characterized by tardy emissions, sometimes none at all. This condition has been called aspermatism. The following cases exemplify this state of affairs: A shoemaker, 20 years old, has masturbated, on an average, once every night from his fifteenth year up to three weeks ago, when he became alarmed at reading a book on self-abuse, which had fallen into his hands, since which time he has abandoned the habit. For the past eighteen months he has noticed in masturbating that it required at least five minutes to produce an emission, and six months ago, on having sexual intercourse, ejaculation did not recur for quite half an hour. He had a stricture 5½ back, calibre 19, with hyperæsthesia of the canal. I have notes of other cases of this class, but I will say that two out of every three cases of this kind are due to masturbation. I had intended to show you a colored man, a teacher, aged 22 years, who had no ejaculations during sexual congress: he would frequently continue the act of copulation for half an hour and longer without an emission occurring; but, hearing that I would expose him betore the class, he disappeared. In the case of a gentleman from New York, no matter how long he remained or persisted in the venereal act, he could not have an emission. These cases show diminished reflex spinal excitability, and should receive your earnest attention.

No book tells you how to treat these cases, and I take not a little credit to myself in having first distinctly and clearly pointed them out. I have repeatedly stated to you that stricture is a not infrequent consequence of masturbation, and I framed my assertion upon personal observation, which taught me that these strictures were of large calibre, and in 77 per cent. single, 15 per cent. were in the first inch of the urethra. In order to exiend my experience, I visited the Almshouse and Dr. Kirkbride's. From the experience and information these investigations gave me, I read a paper in Chicago, in which I cited cases as follows: All the subjects were too young when admitted to the children's asylum to have acquired gonorrhœa, and were removed from any possible contact with females; they were found, those who were idiotic, insane or epileptic, in the insane department under constant surveillance, yet, in spite of all, they were prodigious masturbators. Exploration of their urethræ showed strictures and marked hypræsthesia. The use in knowing that stricture can and does follow onanism has a practical bearing, for you will not be led to believe that because a patient tells you he never had gonorrhœa that he has no stricture. How can you cure these cases? Remove the causes, overcome the hyperæsthesia, cure the stricture, and correct the constitutional abnormalities. For three purposes we employ bromide of potassium: to overcome the acridity of the urine, obtund the sensibility of the mucous membrane of the urethra, and keep the sexual desire, for the time being, in abeyance. The bromide of camphor is also a valuable drug. Let the patient avoid women, and tell him not to excite his sensual apppetite; let him abstain from malt liquors; but if he must drink, a small quantity of whisky or port is less injurious. He is to sleep on a hard mattress, and relieve his bladder as soon as cognizant of its distension. Horseback exercise and riding in carriages or cars must be avoided. The introduction of bougies is the best local treatment. Every fourth day the bougie should be inserted, and at the end of four introductions a size larger may be employed, and it should remain a little longer in the canal. If a stricture exists, remove it by an incision. Sometimes functional heart troubles complicate your case; here digitalis, in an infusion, which I believe is the most reliable way to give it, or digitalin, 1-60th gr., may be ordered as often as the case demands. When the subject is plethoric, give him the antimonial and saline mixture; but when he is anæmic build him up. Never use aphrodisiacs. Cantharides, damiana, belladonna, have an action similar to alcohol; the secondary depressant action of these agents exerts a most pernicious effect upon your case. Guard your patient against experimenting; and, if he is about to marry, let him be careful not to exceed a very moderate number of sexual performances. Having now finished the motor, sensory, and secretory neuroses resulting from urethral disease, we will proceed to the study of the venereal affections.—*Medical Bulletin.*

